# Case Report: Mucous Membrane Pemphigoid With IgG and IgA Anti-Laminin γ1 Antibodies and IgA Anti-Laminin α5 Antibodies

**DOI:** 10.3389/fimmu.2022.903174

**Published:** 2022-06-01

**Authors:** Wenjing Kuang, Hua Qian, Qiyue Zhang, Wei Li, Takashi Hashimoto, Xin Zeng, Xiaoguang Li

**Affiliations:** ^1^State Key Laboratory of Oral Diseases, National Clinical Research Center for Oral Diseases, Chinese Academy of Medical Sciences Research Unit of Oral Carcinogenesis and Management, West China Hospital of Stomatology, Sichuan University, Chengdu, China; ^2^Dermatology Hospital of Jiangxi Province, Jiangxi Provincial Clinical Research Center for Skin Diseases, Candidate Branch of National Clinical Research Center for Skin Diseases, Dermatology Institute of Jiangxi Province, The Affiliated Dermatology Hospital of Nanchang University, Nanchang, China; ^3^Department of Dermatovenereology, Rare Disease Center, West China Hospital, Sichuan University, Chengdu, China; ^4^Department of Dermatology, Osaka Metroplitan University Graduate School of Medicine, Osaka, Japan

**Keywords:** mucous membrane pemphigoid, laminin γ1, laminin α5, autoantibody, oral lesion

## Abstract

Mucous membrane pemphigoid (MMP) and anti-laminin (LM) γ1 pemphigoid, two subtypes of subepidermal autoimmune bullous diseases characterized by autoantibodies against epidermal basement membrane zone proteins, mainly show mucosal and skin lesions, respectively. The known autoantigens of MMP includes BP180, BP230, LM332, integrin α6β4 and type VII collagen, and anti-LMγ1 pemphigoid targets LMγ1. In this study, we present an unique MMP case with oral mucosal lesions, which showed positive IgA signals on basement membrane zone in indirect immunofluorescence using normal human skin and on dermal side in indirect immunofluorescence using salt-split skin, positive IgA autoantibodies against LMγ1 by immunoblotting of epidermal extracts, positive IgA autoantibodies against LMα5 by immunoblotting of LM521 recombinant protein (rLM521) and positive IgG autoantibodies against LMγ1 by immunoblotting of rLM111 and rLM521 at first visit (Day 0). After therapy, further serological analyses of serum samples collected at Day 30 and Day 50 indicated that IgA autoantibodies against LMγ1 were likely to be pathogenic. These results suggest that LMγ1 is another autoantigen of MMP, and our patient might be the first reported case of anti-LMγ1 MMP.

## Introduction

Subepidermal autoimmune bullous diseases are a group of rare skin disorders characterized by autoantibodies against epidermal basement membrane zone (BMZ) proteins which include bullous pemphigoid, mucous membrane pemphigoid (MMP), anti-laminin (LM) γ1 pemphigoid and others (1).

MMP affects one or more mucous membranes and occasionally involves the skin (1). Known MMP-related autoantigens are BP180, BP230, LM332, integrin α6β4 and type VII collagen (2).

Anti-LMγ1 pemphigoid, also called anti-p200 pemphigoid, was first reported in 1996 as unique two cases with IgG autoantibodies to an unidentified 200-kDa protein (p200) present at the dermal side of BMZ (3). In 2009, our group identified this p200 autoantigen as LMγ1 and therefore proposed to rename this disease entity as anti-LMγ1 pemphigoid (4). Clinically, anti-LMγ1 pemphigoid presents mainly skin lesions and occasionally mucosal lesions (5).

In this study, we present a unique MMP case with only oral mucosal lesion, in which our serological studies suggested IgA anti-LMγ1 autoantibodies as pathogenic antibodies. We finally made a tentative diagnosis of anti-LMγ1 MMP for this case.

## Case Description

**Figure 1:**
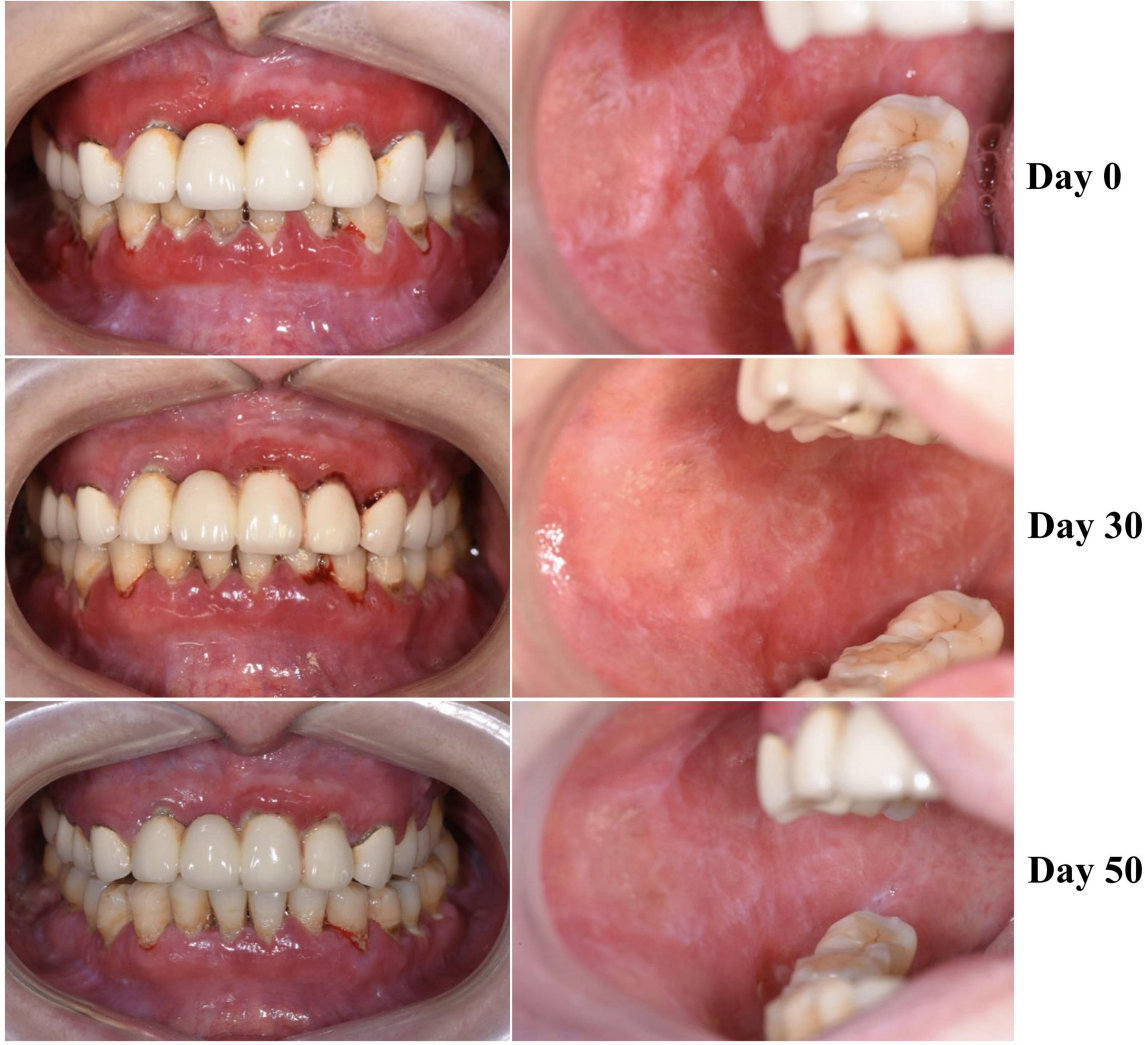
Changes of clinical features of this patient. The clinical features on the gingivae (left) and buccal mucosa (right) at Days 0, 30 and 50 are shown.

**Figure 2:**
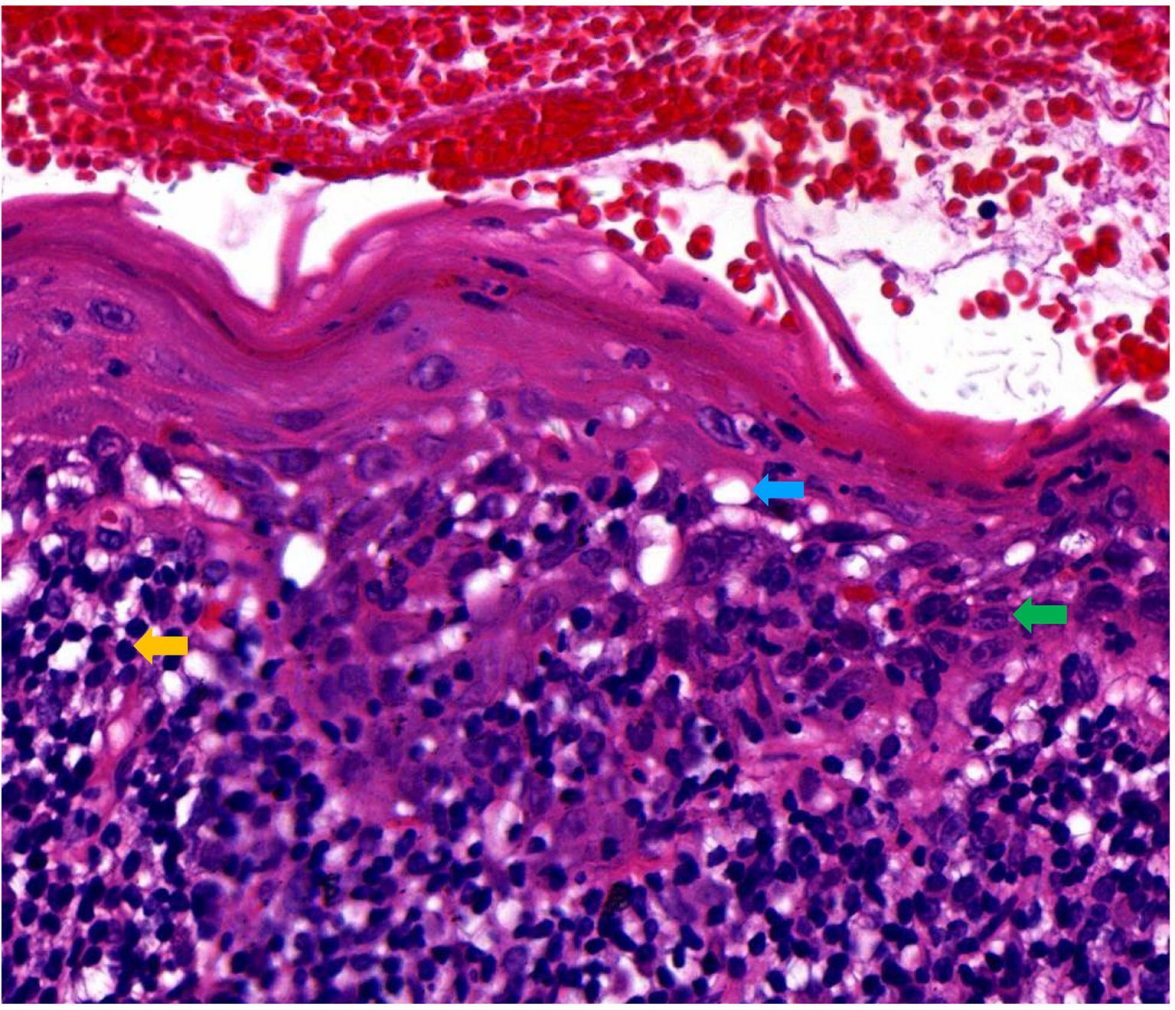
Histopathological features of this patient. Histopathological features for the biopsy taken from the lesions on the left buccal mucosa (H&E staining, original magnification, x200). Blue, green and yellow arrows indicated the vacuolar degeneration, plasma cell and lymphocyte, respectively.

A 49-year-old female presented with erythematous and erosive oral mucosal lesions on the gingiva, tongue and buccal mucosa, and white striae on the tongue and buccal mucosa (**Figure 1**, Day 0) without any lesions on the skin or other mucous membranes Histopathology of biopsy from white striae on the left buccal mucosa showed atrophic mucosal epithelium with vacuolar degeneration of basal cells, and severe inflammatory infiltration of lymphocytes and plasma cells with formation of lymphoid follicle in the upper dermis (**Figure 2**). Direct immunofluorescence showed no BMZ deposition for IgG, IgA, IgM and C3 (data not shown).

**Figure 3:**
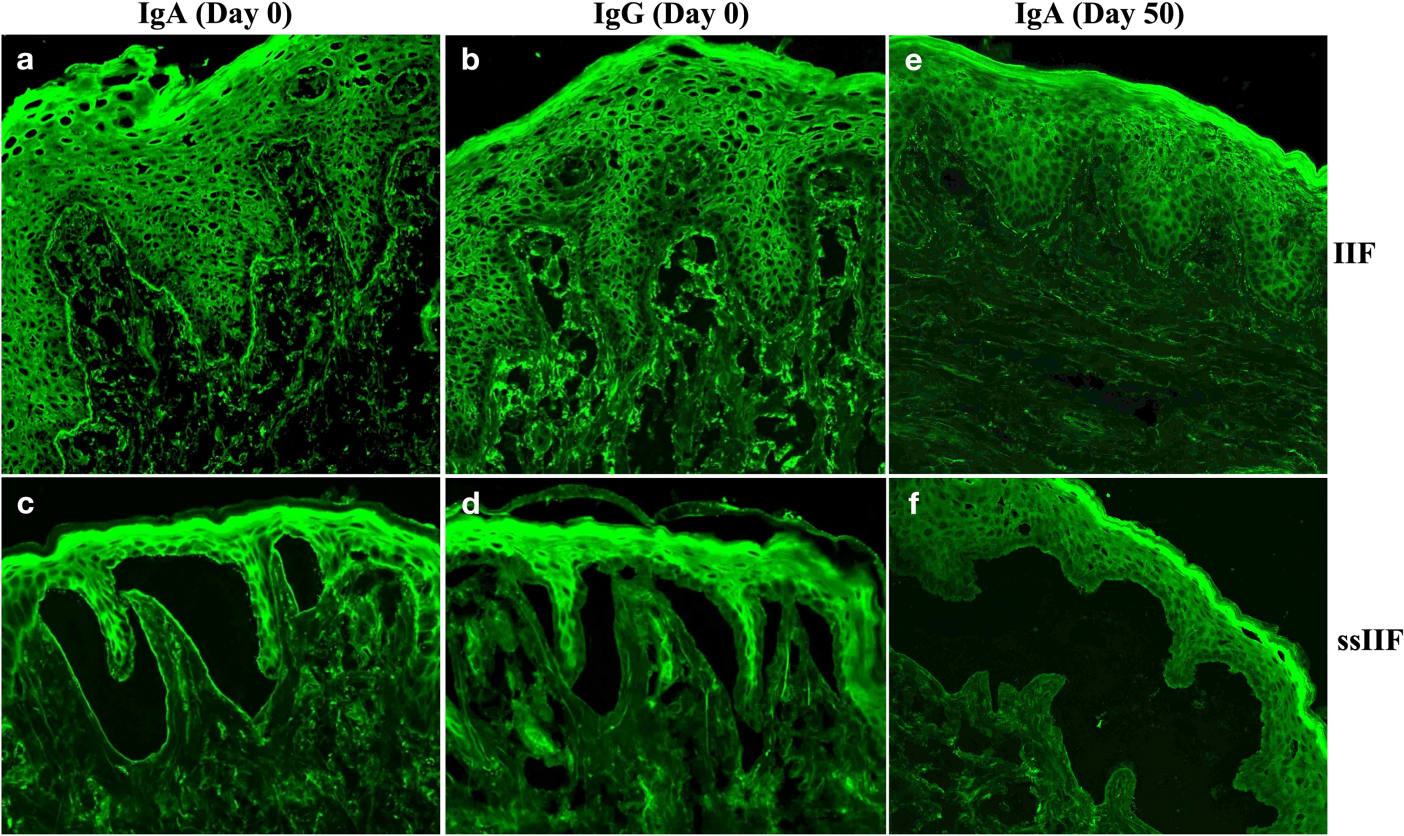
Indirect immunofluorescence using normal human skin (IIF) and using 1M NaCl-split normal human skin (ssIIF) for the patient serum taken at Day 0 and Day 50. IgA **(A)**, but not IgG of **(B)**, anti-BMZ antibodies were detected in IIF for serum taken at Day 0. IgA **(C)**, but not IgG **(D)** antibodies bound to the dermal side of the split in ssIIF for serum taken at Day 0. IgA antibodies were not detected in both IIF **(E)** and ssIIF **(F)** for serum taken at Day 50.

By indirect immunofluorescence (IIF) using normal human skin, the patient serum taken at Day 0 showed IgA, but not IgG, anti-BMZ antibodies, without antibodies to keratinocyte cell surfaces (**Figures 3A, B**). By IIF using 1M NaCl-split normal human skin (ssIIF), the patient serum taken at Day 0 showed only IgA binding to the dermal side of the split skin (**Figures 3C, D**).

**Figure 4:**
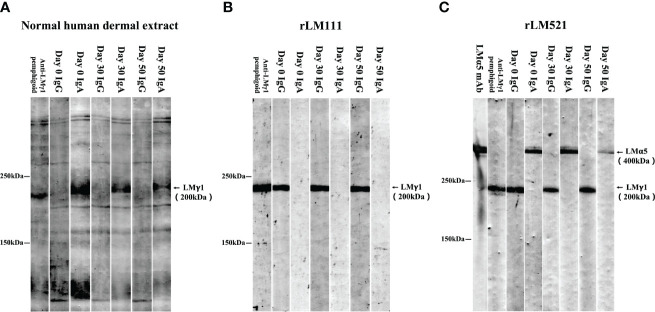
Immunoblotting analyses. Immunoblotting analyses of normal human dermal extract **(A)**, rLM111 **(B)** and rLM521 **(C)** were performed for IgG and IgA autoantibodies against LMγ1 and LMα5 in patient sera collected at Days 0, 30 and 50. The serum of an anti-LMγ1 pemphigoid patient and anti-LMα5 monoclonal antibody (LMα5 mAb) were used as positive controls.

We next performed various immunoblotting and ELISA assays for this patient serum taken at Day 0. Immunoblotting of normal human dermal extract detected IgA, but not IgG, autoantibodies against LMγ1 (**Figure 4A**). Immunoblotting of LM111 recombinant protein (rLM111) detected IgG, but not IgA, autoantibodies against LMγ1 (**Figure 4B**). Furthermore, IgA anti-LMα5 and IgG anti-LMγ1 autoantibodies were identified in immunoblotting of rLM521 (**Figure 4C**). This serum showed negative results in other tests, which are routinely performed in our laboratory for detection of other known autoantibodies in autoimmune bullous diseases, including commercially available ELISAs for BP180, BP230, type VII collagen, desmoglein 1 and desmoglein 3 (MBL, Nagoya, Japan), and in house ELISAs using recombinant proteins of LM332, integrin α6β4, integrin β4 intracellular domain, integrin β4 extracellular domain, peptides of BP180, desmoglein 1 and desmoglein 3, as well as immunoblotting analyses of normal human epidermal extract, concentrated culture supernatant of HaCaT cells and hemidesmosome-rich fraction prepared from A431 cells.

Based on the clinical features and immunological findings, this patient was first diagnosed as MMP with IgG and IgA autoantibodies against LMγ1 and IgA antibodies against LMα5. The treatment with minocycline hydrochloride 100 mg/day was initiated.

Then, this patient visited us two more times at Day 30 and Day 50. Clinically, erosive oral mucosal lesions improved with time on the therapy (**Figure 1**, Day 30 and Day 50). By both IIF using normal human skin and ssIIF, neither IgG nor IgA anti-BMZ antibodies were detectable for sera collected at Day 30 and Day 50 (**Figures 3E, F** and data not shown). In immunoblotting of normal human dermal extract, IgA reactivity with LMγ1 reduced gradually with time (**Figure 4A**). In immunoblotting analyses of rLM111 and rLM521, IgG reactivities with LMγ1 were not changed with time (**Figures 4B, C**). In immunoblotting of rLM521, IgA reactivity with LMα5 at Day 30 was similar to that at Day 0, but reduced at Day 50 (**Figure 4C**).

Based on the changes of clinical features, IgA reactivity in IIF studies, and the results of the 3 immunoblotting studies along with time after the therapy, we considered that IgA, but not IgG, autoantibodies against LMγ1 may be pathogenic, while it is currently unknown whether circulating IgA autoantibodies against LMα5 were involved in the pathogenesis of this disease. Therefore, we finally made a tentative diagnosis of anti-LMγ1 MMP.

## Discussion

In a very recent MMP study for 154 cases, the rates of autoantigen detection for BP180, BP230 and LM332 were 58.4%, 1.9% and 8.4%, respectively (6). In 2017, we reported 721 MMP suspected patients, in which the rates of autoantigen detection for BP180 alone, LM332 alone and both of BP180 and LM332 were 38.5%, 20.9% and 7.5%, respectively (7). In another MMP study for 78 cases, the rates of autoantigen detection for BP180, BP230, LM332 and type VII collagen were 33.3%, 11.5%, 11.5% and 3.8%, respectively (2). Although large cohort studies of antibodies to integrin α6β4 in MMP have not yet been performed, we reported that 27 (62.8%) of 43 pure ocular MMP patients targeted integrin β4 (8). The results of these studies suggests that BP180 is the major autoantigen of MMP, followed by LM332 and integrin α6β4, whereas some unidentified autoantigen(s) may still exist.

The present study suggested that LMγ1 is also an autoantigen of MMP. Anti-LMγ1 antibodies were detected in previously reported MMP cases with known MMP autoantibodies (9). However, our patient was the first case with clinical features of MMP, which showed antibodies to LMγ1 (and LMα5) but not any known MMP autoantibodies, suggesting that LMγ1 is one of the MMP autoantigens. Therefore, it is necessary to screen autoantibodies against LMγ1 in MMP suspected cases, particularly when autoantibodies against BP180, LM332, integrin α6β4, BP230 and type VII collagen were negative. Our case is also the first case with IgA antibodies to LMα5, although their pathogenic relevance is currently unclear and future studies for the similar cases are needed to clarify it.

For detection of IgG autoantibodies against LMγ1 in the sera of anti-LMγ1 pemphigoid cases, we usually use immunoblotting analyses of normal human dermal extract and LMγ1-contaning LM trimer such as rLM521 and rLM111, and all of these tests are positive in most cases (4, 9). In addition, anti-LMγ1 pemphigoid cases rarely show IgA anti-LMγ1 antibodies (5). However, although all these immunological studies were repeatedly performed in our anti-LMγ1 MMP case, immunoblotting of dermal extract always detected IgA anti-LMγ1 autoantibodies, and those of rLM521 and rLM111 detected IgG anti-LMγ1 antibodies. The true reason for this discrepant reactivity with LMγ1 is currently unknown. However, the different reactivity of IgG and IgA antibodies may be due to the different epitopes within LMγ1 molecule for IgG and IgA antibodies.

On clinical features, our anti-LMγ1 MMP case showed only oral lesions without skin lesions. In contrast, anti-LMγ1 pemphigoid mainly shows skin lesions with occasional concurrence of oral mucosal lesion.

Considering the positive IgA anti-BMZ antibodies detected by IIF and ssIIF, the negative results of direct immunofluorescence might be false negative. For diagnosis of autoimmune bullous diseases, there is a certain false negative rate of direct immunofluorescence, particularly for MMP. Actually, in our recently published paper, the positive rate of direct immunofluorescence in anti-LM332-type MMP was 89.8% (88 in 98 cases) (10).

There is a limitation of this study that minocycline hydrochloride might simply be acting as a protease inhibitor, and therefore serum autoantibody titers might not be correlated with disease response.

In summary, we reported for the first time that LMγ1 may also be another autoantigen of MMP, and propose a new disease entity, anti-LMγ1 MMP. Significance of IgA antibodies to LMα5 should be clarified in the future studies for the similar cases.

## Data Availability Statement

The original contributions presented in the study are included in the article/supplementary material. Further inquiries can be directed to the corresponding authors.

## Ethics Statement

The studies involving human participants were reviewed and approved by Dermatology Hospital of Jiangxi Province. The patients/participants provided their written informed consent to participate in this study. Written informed consent was obtained from the individual(s) for the publication of any potentially identifiable images or data included in this article.

## Author Contributions

All authors were involved in drafting the article or revising it critically for important intellectual content, and all authors approved the final version to be published. WK, XZ, and XL had full access to all of the data in the study and took responsibility for the integrity of the data and the accuracy of the data analysis. TH, XZ, and XL conceived and designed the project. WK, HQ, QZ, WL, TH, XZ, and XL collected case information and laboratory data, and analyzed the data. All authors contributed to the article and approved the submitted version.

## Funding

This work was supported by the National Natural Science Foundation of China (NSFC) (Grant Nos. U19A2005) and CAMS Innovation Fund for Medical Sciences (CIFMS) (Grant Nos. 2020-I2M-C&T-A-203).

## Conflict of Interest

The authors declare that the research was conducted in the absence of any commercial or financial relationships that could be construed as a potential conflict of interest.

## Publisher’s Note

All claims expressed in this article are solely those of the authors and do not necessarily represent those of their affiliated organizations, or those of the publisher, the editors and the reviewers. Any product that may be evaluated in this article, or claim that may be made by its manufacturer, is not guaranteed or endorsed by the publisher.
